# Contactless radar-based heart rate estimation in palliative care – a feasibility study and possible use in symptom management

**DOI:** 10.1186/s12904-024-01592-3

**Published:** 2024-11-30

**Authors:** Stefan G. Grießhammer, Anke Malessa, Hui Lu, Julia Yip, Julie Leuschner, Florian Christgau, Nils C. Albrecht, Marie Oesten, Thanh Truc Tran, Robert Richer, Maria Heckel, Bjoern M. Eskofier, Alexander Koelpin, Tobias Steigleder, Christoph Ostgathe

**Affiliations:** 1 Department of Palliative Medicine, Universitätsklinikum Erlangen, Friedrich-Alexander-Universität Erlangen-Nürnberg (FAU), Erlangen, Germany; 2 Institute of High-Frequency Technology, Hamburg University of Technology (TUHH), Hamburg, Germany; 3 Chair of Electronics and Sensor Systems, Brandenburg University of Technology Cottbus-Senftenberg, Cottbus, Germany; 4 Machine Learning and Data Analytics Lab, Friedrich-Alexander-Universität Erlangen-Nürnberg (FAU), Erlangen, Germany

**Keywords:** Palliative Care, Symptom management, Radar technology, Vital signs, Machine learning, Artificial intelligence

## Abstract

**Background:**

Heart rate (HR) monitoring is a medical standard to provide information about a patient’s health status. In palliative care, relationship and social engagement are crucial therapeutic concepts. For fear of disrupting communication, social contact, and care, continuous HR monitoring is underutilised despite its potential to inform on symptom burden and therapeutic effects. This study investigates radar-based HR monitoring as an innovative and burden-free approach for palliative care patients, compares its accuracy with conventional ECG methods, and shows potential for therapeutic guidance.

**Methods:**

A single-centre, comparative clinical trial was conducted with palliative care patients at the ward of the Department of Palliative Medicine of the University Hospital of Erlangen. The HR measurements obtained with radar were compared with Holter ECG (study arm I, overnight) and Task Force^®^ Monitor (TFM)-based ECG validation recordings (study arm II, one hour). In addition, long-term radar measurements without validation were analysed in comparison with clinical health records (study arm III).

**Results:**

Both validation methods showed correlation by scatter plot, modified Bland-Altman plot, and equivalence testing. *N* = 34 patients participated in study arm I. HR of 4,079 five-minute intervals was analysed. Radar measurements and ECG showed high agreement: difference of HRs was within $\:\pm\:$5 bpm in 3780 of 4079 (92.67%) and within ±13.4 bpm ($\:\pm\:$1.96 times the SD of the mean) in 3979 (97.55%) intervals, respectively. In study arm II, *n* = 19 patients participated. 57,048 heart beats were analysed. The HR difference was within $\:\pm\:$5 bpm for 53,583 out of 57,048 beats (93.93%) and within $\:\pm\:$8.2 bpm ( ± 1.96 times the SD of the mean) in 55,439 beats (97.18%), respectively. Arm III showed HR changes extracted from radar data in correlation with symptoms and treatment.

**Conclusion:**

Radar-based HR monitoring shows a high agreement in comparison with ECG-based HR monitoring and thus offers an option for continuous and above all burden-free HR assessment, with the potential for use in symptom management in palliative care, among others. Further research and technological advancements are still necessary to fully realize this innovative approach in enhancing palliative care practices.

## Introduction

Monitoring of heart rate (HR) from palpation to electrocardiography (ECG) plays a crucial role in healthcare. It provides insight into the patients’ state of health. It reflects various aspects of health, from basic cardiovascular function to cardiovascular and systemic diseases, and even complex reactions such as pain or anxiety are reflected in HR and the modulation of heartbeat [1]. Cardiac activity is closely linked to life and its cessation means death [2]. Due to this pivotal role, HR monitoring may impact relationship aspects of care, drawing attention to the mere biomedical perspective.

In end-of-life and palliative care (PC), relationship and social inclusion are key concepts in the therapeutic process. Therefore, PC has traditionally focused more on the interpersonal domain and less on medical technology, like continuously monitoring of HR or other vital signs. In emergency medicine or intensive care constant observation is essential to immediately treat emergencies. Additionally, vital sign monitoring can deliver information on state of health, life-time-prognosis, and symptom burden as well as state of well-being. In PC, this is not intended to initiate emergency measures, but to respond with symptom management, communication, and care. In cancer patients, cardiovascular parameters like ECG-derived HR and blood pressure are already used to monitor and detect physiological responses to pain [3]. Traditional methods, such as ECG, can be intrusive and potentially disruptive, which is contrary to the fundamental principles of PC that emphasize comfort, dignity, and minimal disruption. Hence, monitoring in a PC setting would have to be carried out in a way that these aspects are not compromised.

In PC and medicine in general, there is currently no established option to record HR in an unobtrusive manner. Various approaches have been made to obtain cardiac vital signs in a contactless way, albeit not in a PC scenario. Good results were achieved by electro-optical approaches such as remote photoplethysmography [4], thermography, or infrared technologies [5, 6]. Nevertheless, there are major limitations: necessity of an unobstructed line-of-sight onto the patients’ skin and lighting within a specific range. The necessity for line-of-sight restricts patients’ freedom or results in incomplete data collection. Privacy issues arise as people will be recorded in a video-like manner [7], which has legal implications and affects relationship and care, hindering implementation in PC. A promising alternative to monitor heart action is ballistocardiography, driven also by latest technological developments including the development of small and lightweight inertial measurement unit based sensors on the hardware side [7] and advanced machine learning-based data extraction algorithms on the software side [8]. The limitations of ballistocardiography lie in its susceptibility to any movement in and around the bed that could lead to a distortion of the cardiac signal and the variety of movements that overlay the cardiac signal. Even though this monitoring technology is classified as contactless, the patient’s body must still touch the surface to which the ballistic sensors are attached.

An innovative alternative is radar-based monitoring. Radar (Radio Detection and Ranging) systems emit electromagnetic waves that are reflected by objects where the permittivity changes. Permittivity is a measure of how easily a material allows electric fields to pass through. For example, waves move from air through clothing and bedding to be reflected on the surface of the body [9]. The reflected wave is then detected by the radar’s antenna. By overlaying the emitted and reflected waves, an interference pattern is created to compute the displacement between the radar antenna and the surface from which the waves were reflected. This approach, which is called interferometry, allows to record displacement in the magnitude from metres to micrometres, depending on the wavelength of the radar waves [10]. As cardiac signals, such as pulse waves and heart sounds, generate small movements in the micrometre range on the body surface, these displacements between body surface and radar antenna can be detected by the radar system along with all other types of movements [11]. As both the pulse wave and the heart sounds exhibit specific patterns, algorithms can be developed that extract these specific patterns from the comprehensive movement data recorded by the radar [12, 13], allowing to subsequently compute the HR from the extracted interbeat intervals (IBIs) [14, 15]. The radar-based approach has various advantages: radar waves are able to penetrate objects of lower permittivity, e.g., clothes, bedding, and mattresses, with almost no loss of signal amplitude and without requiring line-of-sight [16]. This allows to place radar systems underneath the slatted frame of a patient bed, providing complete freedom of movement for the patient while ensuring that monitoring remains unobtrusive and unnoticed by patient, visitors, or personnel. Additionally, the technology is scalable to higher degrees of freedom, so that monitoring can take place in the whole room without any restrictions of movement to the patient [17]. The risk of electromagnetic interference between the radar-based system and existing medical devices in the ward environment is negligible due to the low transmission power of 100 mW at maximum and the relatively high frequency of 24 GHz, which leads to a low range of the signal. Furthermore, medical devices must be immune to interference from electromagnetic waves of much higher frequencies and field strengths than those reached by the radar system used in this study, according to the IEC 60601-1-2 standard.

Building on our previous work with radar-based vital parameter recording in healthy test subjects [9, 18], this paper presents the first feasibility exploration of a radar-based approach to monitor HR in PC patients. To ensure the accuracy and reliability of the radar-based measurements, we validated the data with a ground truth derived from gold standard methods. Additionally, we present real-life data on how radar-based cardiac monitoring may guide therapeutic decisions in a PC setting.

## Methods

### Study design and setting

This project was designed as a single-centre, comparative clinical trial to compare radar-sensed HRs with HRs derived from the ECG gold standard recordings. The study has been registered in the central study register of the Bavarian Cancer Research Center (BZKF) and can be accessed there under the acronym GUARDIAN [19]. Study duration was 20 months. Individuals willing to participate were able to choose between three study arms: study arm I, being measured by radar and Holter ECG overnight (medium-term validation); study arm II, being measured by radar and the CNSystems Task Force^®^ Monitor (TFM) for one hour (short-term validation); and study arm III, participating in a long-term radar measurement without gold standard validation from the time of study enrolment until discharge from the palliative ward or death. Participants could decide to take part in one or more study arms.

The aim of the individual study arms was to investigate the concordance of radar-based and gold standard recordings of heart rate in a clinical real-world setting (arm I), to assess the radar signal characteristics in terms of robustness and concordance (arm II), and to assess the feasibility of employing radar-based HR monitoring to support symptom detection and guide therapeutic decisions (arm III).

In this paper, we focus on the presentation of results of study arms I and II in terms of validation of radar-based cardiac monitoring and give an outlook on potential to guide care from study arm III.

### Participants

Inclusion criteria were age over 18 years, a life-threatening illness requiring hospitalization and written consent from the patient themselves or their legal representative. Patients who had to be isolated due to an infectious disease and patients who were about to die were excluded from the study. Cardiac diseases and conditions did not a priori lead to an exclusion from enrolment.

Gender, age, height, and weight were extracted from the clinical health record (CHR) Soarian^®^ Clinicals (Cerner Health Services, Inc., Version 4.5.200) for each patient.

### Equipment for measurement

**Fig. 1:**
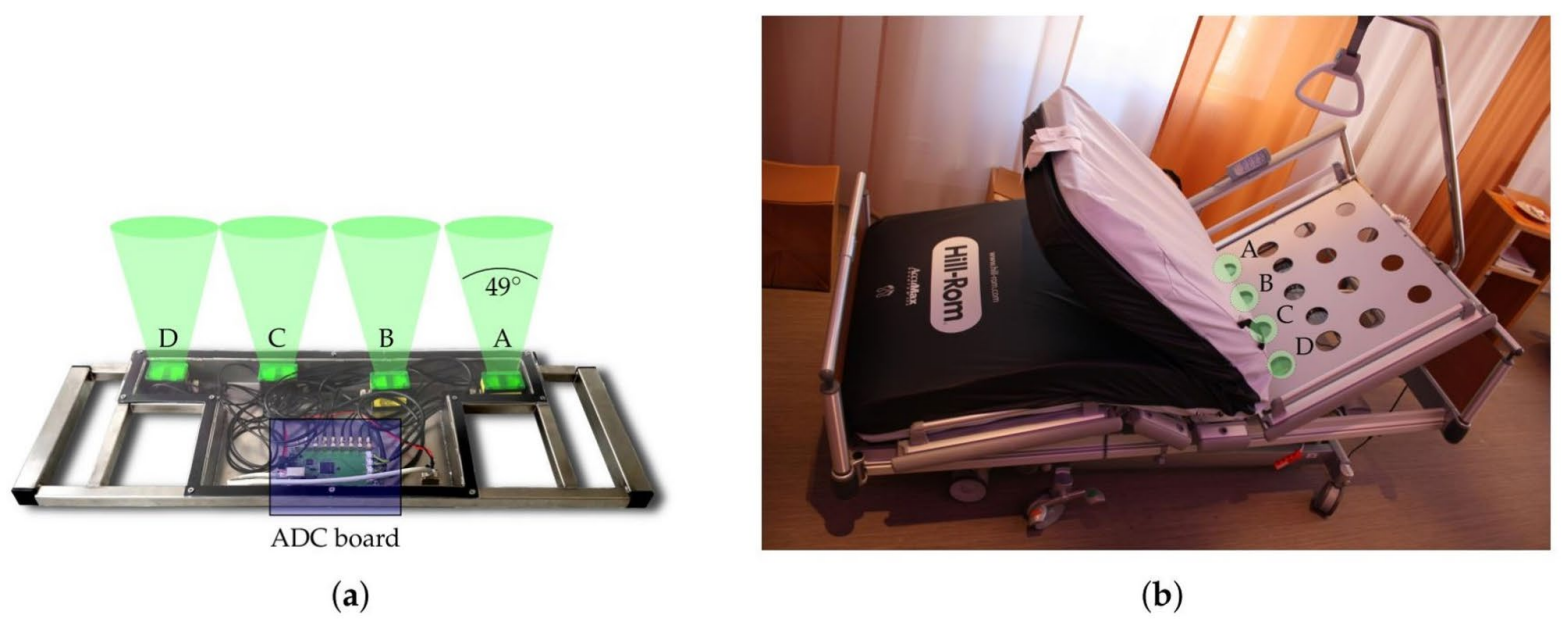
(**a**) Radar system with four continuous-wave radar modules and ADC (Analog to Digital Converters) board. (**b**) Position of the radar modules underneath the head section of the slatted frame (indicated in green). Source: Schellenberger et al. 2020 [11]

In all three study arms, commercially available continuous-wave radar units (iSYS-4001, InnoSenT GmbH, Donnersdorf, Germany) operating within the 24-GHz industrial, scientific, and medical (ISM) frequency band as described by Michler et al. were used for radar measurements [16]. For the conducted measurements four of these radar units were mounted next to each other on a metal bracket at a distance of about 18 cm between each other [16]. The radar modules were positioned underneath the head section of the slatted frame of the hospital bed, approximately at the level of the patients’ chests, and beneath the holes in the frame to ensure minimal signal loss (Fig. 1). All beds (*n* = 12) in the PC unit were equipped with radar systems as described above to ensure that the patient did not have to be moved or change beds if they consented. In the event of non-participation, the radar module was not switched on.

For medium-term validation (study arm I), mobile 3-channel Holter ECGs (TLC9803, Contec Medical Systems Co., Ltd were used, allowing continuous ECG recordings by five-minute intervals of mean HR for a maximum of 24 h.

For short-term validation (study arm II), the Task Force^®^ Monitor (TFM) 3400i (CNSystems Medizintechnik AG, Graz, Austria) was used.

The TFM allows for recording ECG, impedance cardiography (ICG), oscillometric, and non-invasive continuous blood pressure, and features two external inputs. We recorded all signal types during our study, but present only ECG data. A three-lead ECG records electrical cardiac activity. The TFM recorded raw ECG data for leads 1 and 2 according to Einthoven’s triangle and computes lead 3 as well as augmented limb leads. ECG signal is digitized at 1000 Hz with an accuracy of ± 5 µV. All data are sampled simultaneously and exported to Matlab proprietary files.

### Overnight measurement with mobile Holter ECG - study arm I

For ECG recording in study arm I, we used adhesive electrodes for the chest wall leads V1, V3, and V5 according to Wilson’s central terminal [20]. Additionally, we included a reference electrode (middle of the sternum) and a grounding electrode (right lower abdomen). The Holter ECG data provides mean HRs for time intervals of five minutes.

In order to ensure temporal consistency of radar and Holter ECG, the internal clocks of both systems were synchronized. Radar and Holter ECG measurements were started between 6 and 7 p.m. and stopped between 7 and 10 a.m. on the next morning. During the measurements, the patients were allowed to move, talk, and behave without constraints.

### One-hour measurement with TFM – study arm II

We conducted one-hour measurements with radar and TFM, which are continuously synchronized by a random synchronization sequence [11, 21]. This allowed to retrieve beat-wise synchronized datasets of the radar and TFM measurements.

For this purpose, the output channel of radar module D was used for the transfer of the radar microcontroller sequence to the TFM.

### A priori sample size calculation

The a priori sample size calculation for the Bland-Altman-Plot analysis was performed according to [22]. Based on a pilot study with healthy participants we assumed the standard deviation of the differences between measurements by the radar and the ECG to be 4 bpm, while accepting a 95% confidence interval for the limits of agreement of 2.5 bpm. This results in a sample size of 30 patients.

The a priori sample size for the TOST was estimated using the R function sampleN.TOST of the R package PowerTOST [23]. We wanted to reach a power of (1-β) = 0.80, while accepting a type-I error probability of α = 0.05. The equivalence bounds were set to +/- 5 bpm, the standard deviation of the differences between the two measurements was again set to 4 bpm and the expected mean of the differences to 1 bpm as suggested by our pilot study. This resulted in a sample size of 28 patients.

Based on these two sample size estimations and the anticipation of dropouts and technical issues we aimed to include 35 patients in each study arms I and II.

The number of heart rate measurement pairs derived from radar and ECG for each study arm I and II was determined taking into account the constraints of ward routine, staff and technical resources and, most importantly, the potential burden of our study on our patients.

In study arm I, we decided to perform the measurements overnight to ensure that the patients spent most of the time in bed with the radar mounted below the matrass, without restricting their daytime activities. In study arm II, we set the measurement time for each patient to one hour to save staff resources and limit patient burden.

### Data processing

Raw data from the radar system were processed with a customized MATLAB script (Version R2020b, MathWorks©, Natick, MA, USA) [11, 24, 25]. For study arms I and III, intervals were determined during which a person was actually present. This is reflected by the presence value, which is calculated from the input voltage of the reflected radio wave which assumes a value of 1 if the person was in bed, and 0 if the person was not [11]. For all study arms, we extracted the displacement data from the raw radar signals and applied a Butterworth filter filtered in a range from 16 to 80 Hz to obtain the radar heart sound signal. To identify the radar module with the best reliance, a Signal Quality Index (SQI) of the radar heart sound signal was calculated. The SQI contains the signal-to-noise-ratio (SNR) of the radar data as described in [24]. For each 60-second window of radar data, we select the radar module with the highest SQI to determine the HR of the corresponding five-minute interval [11, 24]. This is achieved by applying a hidden semi-Markov model (HSMM) algorithm presented in previous work [20] which detects the first (S1) and second (S2) heart sound in the displacement signal in order to identify heart beats. From the detected heart sounds, we computed the IBIs, defined as the time intervals between two consecutive S1. By averaging the IBIs over time, we obtained the mean HRs: for long term monitoring validation (study arm I) over five-minute intervals [25]; for assessment of the radar signal characteristics (robustness and concordance, study arm II) over ten heart beats; for outlook on the use of monitoring to support symptom detection (study arm III) over 30 heart beats.

The MATLAB script exports a Microsoft Excel (Microsoft^®^ 365, Excel^®^ version 2112) spreadsheet, which contains the respective HR mean values.

**Holter ECG - Study arm I**, raw data were processed using the Software 3 Channels ECG Holter System_TF (OS) (V5.5.2.5, Contec Medical Systems Co., Ltd. 2019).

The mean IBI was calculated for each five-minute interval using the HRV analysis module of the software. Afterwards, we manually transferred the average IBI values to a Microsoft Excel spreadsheet (Microsoft^®^ 365, Excel^®^ Version 2112) with the corresponding time stamps and derived the mean HR.

**TFM - Study arm II**, synchronized radar raw data and TFM data were processed using a modified version of the customized MATLAB script described above.

First, the radar and TFM systems were synchronized by aligning the synchronization sequences from both systems. The SQIs of each radar module were calculated over a period of 3 s. The MATLAB script generated beat-to-beat synchronized radar and TFM data for each patient in an Excel file.

In sections of noisy ECG signals and sections where TFM R-peak detection failed, we manually annotated R-peaks in order to ensure sufficient data quality using the open source data labelling software Label Studio (Version 1.6.0) [26]. Since the algorithm for radar-based heartbeat detection is not trained for arrhythmias, we further annotated supraventricular (SVES) and ventricular extra systoles (VES), as well as other arrhythmias. Two physicians performed the annotations independently. Disagreements were discussed and consensus could be reached in all cases of disagreement. The results of the annotations were extracted and automatically added beat-wise to the Excel file of each patient with the beat-to-beat synchronized radar and TFM data. We excluded the annotated data from analysis as noisy ECG signal sections led to loss of ground truth.

Additional data processing steps were performed with the programming language R (Version 4.3.0) [27] via the integrated development environment RStudio (Version 2023.06.0 Build 421) [28]. For each beat, the radar module with the best reliance in comparison with the ECG’s HR was chosen. Next, we determined the HR employing a rolling mean of 10 IBIs from radar and ECG separately. In cases of low SNR, the HSMM generated false positive heart sounds and/or did not detect heart sounds, thereby effectively doubling or halving the HR. These systematic errors were evaluated by calculating the difference between the rolling means of radar and ECG. When the difference was within ± 10% of the half or double of the mean HR of the ECG, the beat was excluded from the dataset of all patients.

### Long-term radar measurements without gold standard – study arm III

In study arm III, radar data was processed analogously to study arm I with the difference that the radar module with the highest SQI within a 30-second window was selected to determine the HR. A 30-second window was chosen in order to best reflect the dynamics of HR changes due to symptoms and/or medication on the one hand and the robustness of the HR analyses on the other.

In addition, a two-stage outlier detection has been performed aiming to eliminate genuine outliers while preserving valid HR data. First, global outliers were identified and removed based on predefined thresholds to remove physiologically impossible HR values. Subsequently, local outliers were excluded through an iterative process that analyses smaller segments of the HR data to detect inconsistencies within the context of each segment as clinically implausible data. We extracted information on medication and patient-reported symptoms and symptom relief based on the CHR. This comprised symptoms, symptom severity, relief of symptoms based on MIDOS_2 assessment [29], medication, dosage, time, and route of administration.

### Statistical analysis

For statistical analysis, we used SPSS Statistics (version 28, IBM^®^ 2021 and version 29, IBM^®^ 2023) and the programming language R (Version 4.3.0) [27] via the integrated development environment RStudio (Version 2023.06.0 Build 421) [28].

Statistical analysis was carried out for the data from study arm I and II separately, but in the same manner.

As suggested by Müller and Büttner [30], we first carried out a concordance analysis by performing the following steps: Plotting a square scatter diagram, plotting a modified Bland-Altman diagram, performing an equivalence test.

For this, we first transferred the data of all patients from the Excel spreadsheet to SPSS. Afterwards, we plotted the mean HRs of the radar (HR_mean radar_) against the mean HRs of the ECG (HR_mean ECG_), yielding a square scatter diagram.

In addition, we created a modified Bland-Altman plot as described in [31] with HR_mean ECG_ on the x-axis and HR_diff_ = HR_mean radar_ – HR_mean ECG_ on the y-axis.

To test for the equivalence of both measurement methods, a two one-sided test (TOST) according to Schuirmann [32] was performed. Thus, HR_mean radar_ and HR_mean ECG_ values were transferred to RStudio. The upper and lower borders were set as + 5 beats per minute (bpm) and − 5 bpm, respectively. Differences that fall within these borders are considered as small enough to be clinically irrelevant.

The hypothesis tested was

(HR_mean radar_ – HR_mean ECG_) <= -5 bpm or (HR_mean radar_ – HR_mean ECG_) > = 5 bpm.

If the hypothesis is accepted, there is no equivalence between the measurements. If the hypothesis is rejected, the alternative hypothesis

-5 bpm < (HR_mean radar_ – HR_mean ECG_) < 5 bpm

is accepted and it is concluded that the measurements are equivalent.

For the type I error, a significance level of α = 0.05 was chosen.

## Results

### Overnight measurement with radar and Holter ECG validation - study arm I

#### Participants and data evaluation

*N* = 34 patients were included in study arm I. The data of four patients (all male) could not be evaluated due to one of the following reasons: failure of two out of three radar systems (*n* = 1 patient), no radar data recorded (*n* = 2), termination of recording after less than two hours (*n* = 1). Out of the 30 remaining patients, 21 (70%) were female. After exclusion of the non-evaluable cases the average age was 67,9 ± 13,3 years, with a minimum of 39 and a maximum of 95 years.

### Statistical analysis

In total, 4592 five-minute intervals were generated. Of these, 513 intervals (12%) were excluded due to missing radar values, i.e. 4079 valid intervals were included in the analysis. Statistics of the radar and ECG average HRs are listed in Table 1.

**Table 1:** Descriptive statistics of the HR mean values of radar and ECG and the HR difference (HR_mean radar_ – HR_mean ECG_) of the 4079 valid five-minute intervals (HR in bpm)

	Minimum HR [bpm]	Maximum HR [bpm]	Mean HR [bpm]	standard error	standard deviation (σ)	variance (*R*^2^)
Radar	39.5	134.5	79.5	0.251	16.1	258
ECG	47.3	157.9	80.4	0.244	15.6	244

### Concordance analysis

**Fig. 2:**
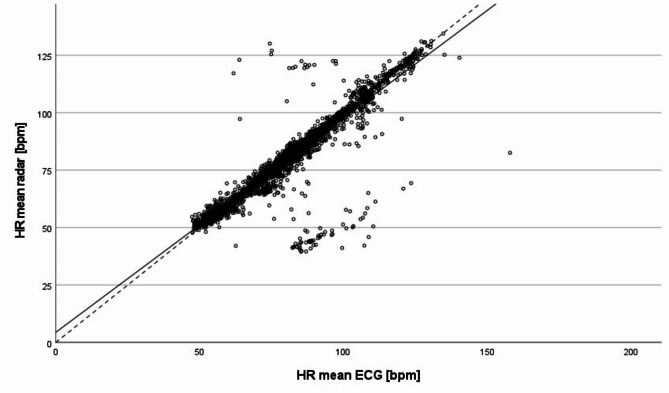
Scatter plot of all 4079 valid five-minute intervals of the overnight measurements: Comparison of the mean HR values of the radar and the Holter ECG; dashed line: angle bisector, solid line: regression line

Fig. 2 shows the scatter plot of all 4079 valid five-minute intervals. It shows that most of the data points are scattered in close proximity to the angle bisector, which is also confirmed by the slope of the estimated regression line (y = 4.4 + 0.93 · x).

**Fig. 3:**
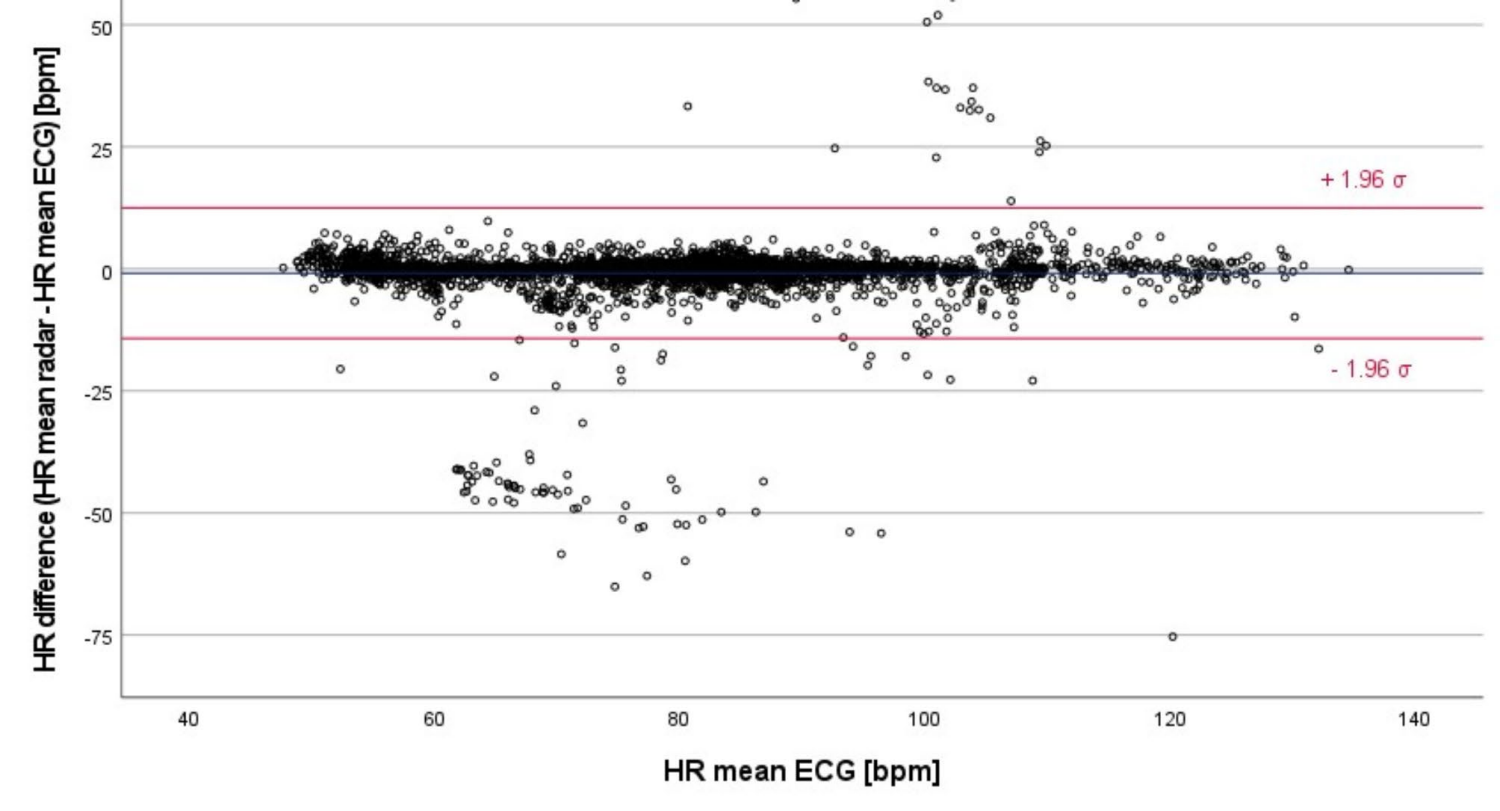
Modified Bland-Altman plot of all 4079 valid five-minute intervals of the overnight measurements – comparison of the mean values of HR_radar_ and HR_ECG_; blue line: overall mean of the HR differences; red lines: (mean of all HR differences) ± 1.96 · σ

Similar trends can be seen in the modified Bland-Altman plot, comparing the five-minutes HR intervals from radar and Holter ECG (Fig. 3).

The overall mean of the HR differences is -0.852 bpm. Furthermore, 3780 of 4079 values (92.67%) lie within $\:\pm\:$5 bpm for 3979 five-minutes intervals (97.55%) the HR difference was equal to or less than ±13.4 bpm ($\:\pm\:$1.96 times the SD of the mean).

### Test for equivalence

The results of the test for equivalence of the data obtained by radar and Holter ECG measurement are summarized in Tables 2 and 3.

**Table 2:** Results of the TOST for overnight measurements

		t	df	*p*
Raw	t-test	7.98	4078	< 0.001
	TOST lower	54.81	4078	< 0.001
	TOST upper	-38.86	4078	< 0.001

**Table 3:** Results of the TOST for overnight measurements: raw effect size

			90% Confidence Interval	
		Estimate	Lower bound	Upper bound
Raw	Equivalence bounds [bpm]		-5	5
	90% confidence interval	0.852	0.676	1.027
Hedges’s g(z)	Equivalence bounds		-0.733	0.733
	90% confidence interval	0.125	0.099	0.151

As shown in Table 3, the 90% confidence interval lies completely within the equivalence bounds for the raw data as well as for Hedges’s g(z). This means that the hypothesis (i.e. null equivalence) is rejected and the alternative hypothesis (i.e. equivalence) is accepted.

### One-hour measurement with TFM-based validation – study arm II

#### Participants and data evaluation

*N* = 19 patients could be included in the study. The data of three patients could not be evaluated due to missing radar data. Furthermore, the TFM data of one patient was compromised and could not be evaluated.

After exclusion, the average age of the remaining 15 patients was 62.9 ± 13.3 years, with a minimum of 38 and a maximum of 83 years, 9 were female.

After annotation of the TFM-derived ECG data we excluded all beats with annotated wrong beat detection, noisy signals, extra systoles, and other arrhythmias. The data of one patient showed a high number of extra systoles (1077 VES, six SVES and three other arrhythmias out of 5573 total recorded beats). Therefore, the IBIs of 2172 beats were affected in total, which corresponds to 39.0% of all heart beats of this patient. In another participant, 849 beats of 4142 beats were affected, which amounts to 20.5% of all heart beats. Therefore, the data of these patients were excluded from the dataset.

In total, our dataset of study arm II consists of 57,048 heart beats from 13 patients which was further analysed.

#### Descriptive statistics

The descriptive statistics values of the heart beats determined by the TFM and the radar (in bpm) can be found in Table 4.

**Table 4:** Descriptive statistics of the 57,048 h values (rolling mean) determined by the TFM and the radar (HR in bpm)

	Minimum HR [bpm]	Maximum HR [bpm]	Mean HR [bpm]	standard deviation (σ)
ECG (TFM)	53.4	129.2	79.0	15.8
Radar	47.6	140.7	78.3	15.3

#### Concordance analysis

**Fig. 4:**
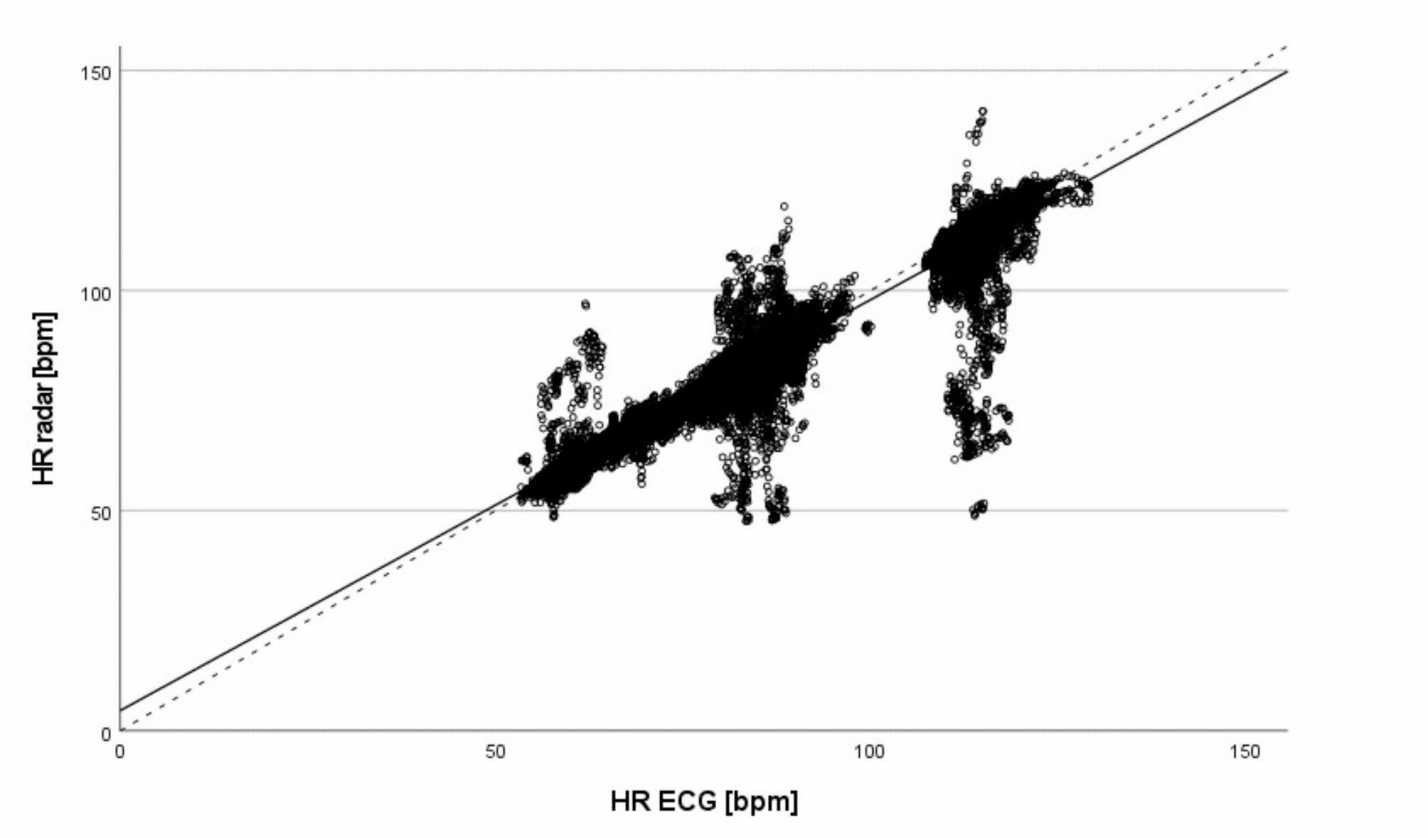
Scatter plot of the rolling mean values of all 57,048 heart beats of the one-hour measurements: Comparison of the HR values (rolling mean over 10 beats) derived from the radar data (HR radar) and from the ECG data of the TFM (HR ECG); dashed line: angle bisector, solid line: regression line

Fig. 4 shows the scatter plot of the rolling mean values of all 57,048 heart beats determined by the TFM and the radar during the one-hour measurements.

As shown in Fig. 4, most of the data points are scattered in close proximity to the angle bisector. The regression line (y = 4.58 + 0.93 · x) is almost identical with the angle bisector.

**Fig. 5:**
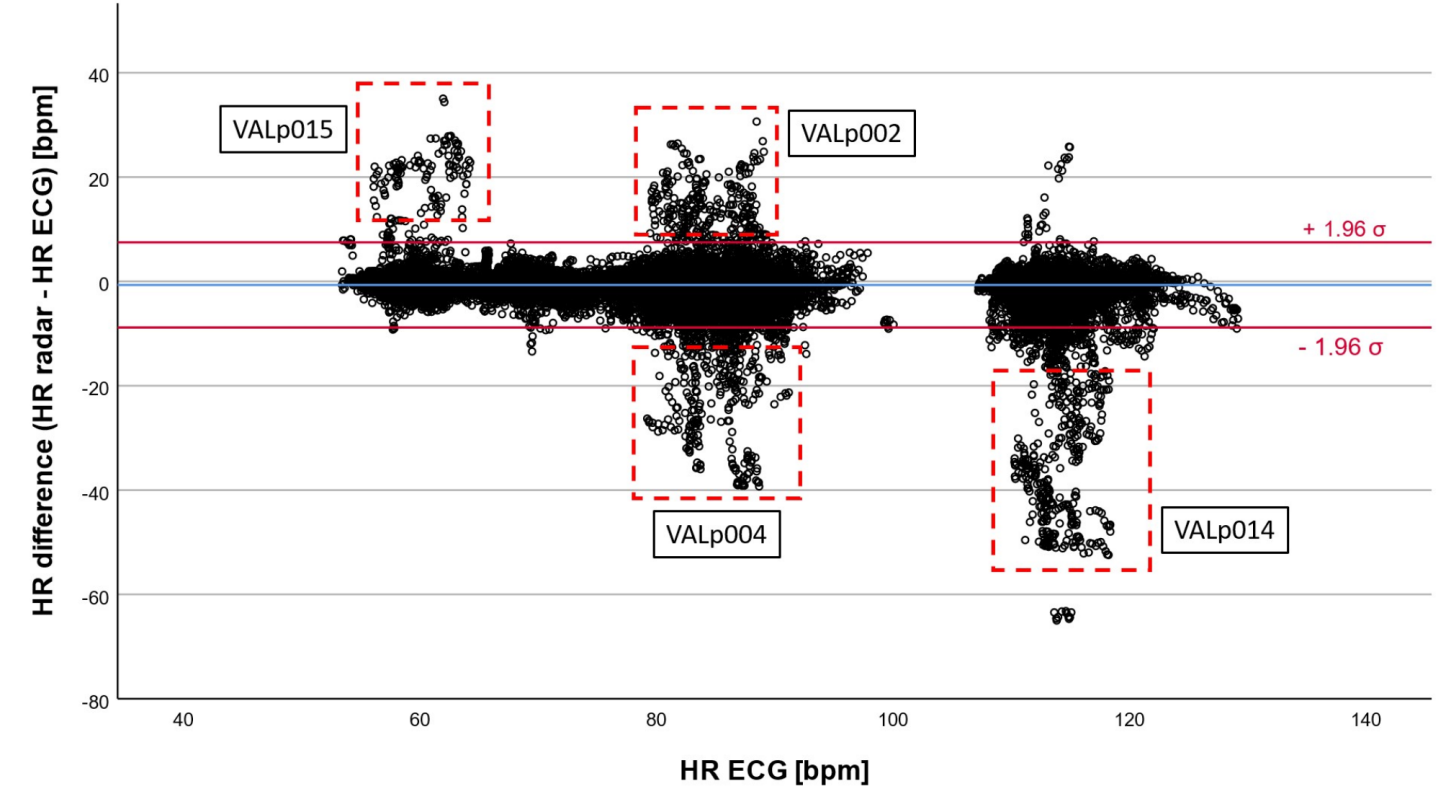
Modified Bland-Altman plot of all 57,048 heart beats of the one-hour measurements – comparison of the rolling mean values of HR_radar_ and HR_ECG_; blue line: overall mean of the HR differences; red lines: (mean of all HR differences) ± 1.96 · σ; red dashed box: outliers related to specific patients (VALp002, VALp004, VALp014 and VALp015), comprising less than 3% of all data points

The modified Bland-Altman plot of all 57,048 heart beats for the comparison between radar- and TFM-derived HR data reveals a mean difference of -0.678 bpm (Fig. 5).

Furthermore, 53,583 out of 57,048 heart beats (93.93%) lie within $\:\pm\:$5 bpm and 55,439 values (97.18%) within $\:\pm\:$8.2 bpm (± 1.96 times the SD of the mean), respectively.

Besides, we identified four clusters of outliers that could be related to the data of four specific patients from our dataset of 13 patients (Fig. 5, red dashed boxes labelled with the patients’ artificial identifiers). These clusters of outliers occur at certain HRs between 55 and 65 bpm, 80 to 90 bpm and 110 to 120 bpm. Although prominent in Fig. 5, these outliers represent less than 3% of the total of 57,048 values. However, since only four of 13 patients account for the majority of these outliers, this finding indicates that the outliers are not evenly distributed across the data of all patients.

#### Test for equivalence

The results of the test for equivalence of the data obtained by radar and Holter ECG measurement are summarized in Tables 5 and 6.

**Table 5:** Results of the TOST for one-hour measurements

		t	df	*p*
raw	t-test	38.91	57,047	< 0.001
	TOST lower	325.74	57,047	< 0.001
	TOST upper	-247.93	57,047	< 0.001

**Table 6:** Results of the TOST for one-hour measurements: raw effect size and Hedges’s g(z)

		Estimate	90% Confidence interval	
			Lower bound	Upper bound
Raw	Equivalence bounds [bpm]		-5	5
	90% confidence interval	0.678	0.650	0.707
Hedges’s g(z)	Equivalence bounds		-1.2	1.2
	90% confidence interval	0.163	0.156	0.170

As shown in Table 6, the 90% confidence interval lies completely within the equivalence bounds. This means that the hypothesis (i.e. null equivalence) is rejected and the alternative hypothesis (i.e. equivalence) is accepted.

#### Long-term radar measurements without validation - study arm III

**Fig. 6:**
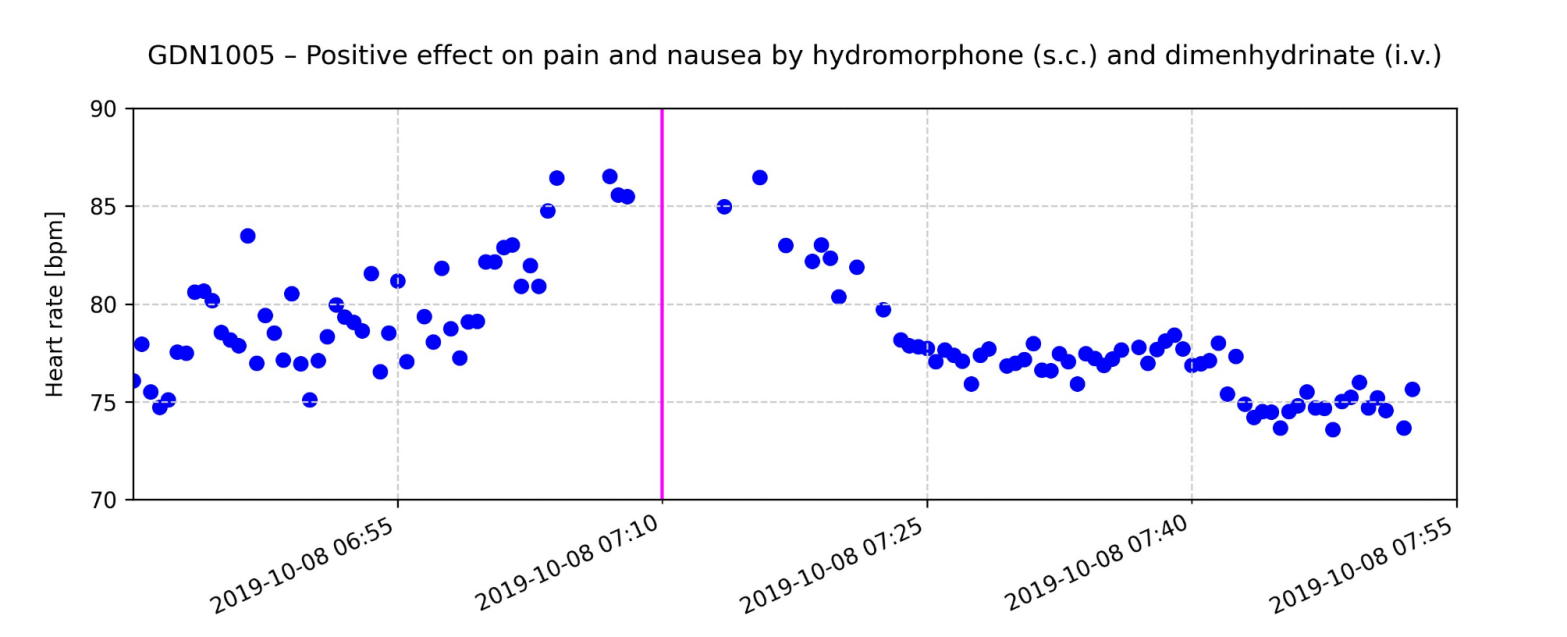
Course of radar-estimated HR over time for one patient; each data point represents the mean HR of a 30-seconds time interval; the pink line indicates the time of administration of 1 mg hydromorphone (s.c.) and 62 mg of dimenhydrinate (i.v.). Clinical health records (CHR) document relief of pain and nausea 20 min after application of medication

**Fig. 7:**
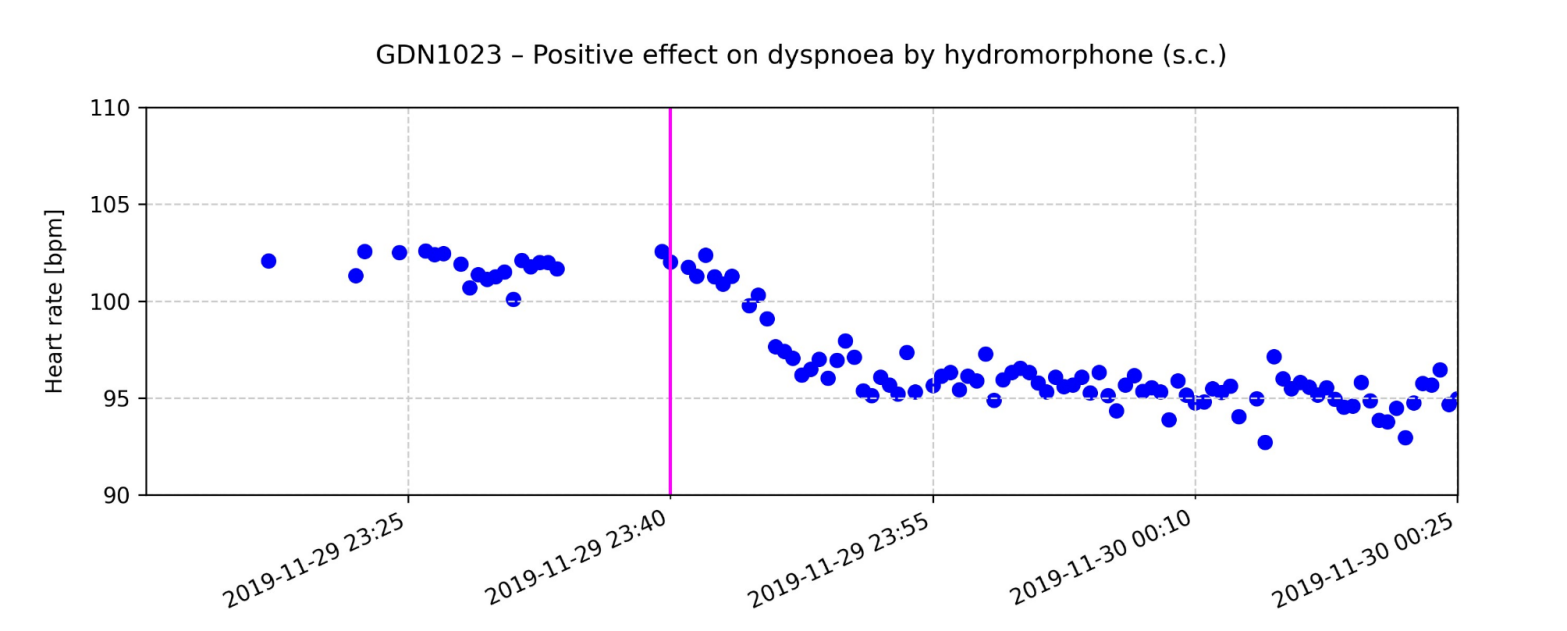
The pink line indicates the time of administration of 0.5 mg hydromorphone (s.c.) for dyspnoea. Clinical health records (CHR) document relief of dyspnoea approx. 30 min after application of medication

**Fig. 8:**
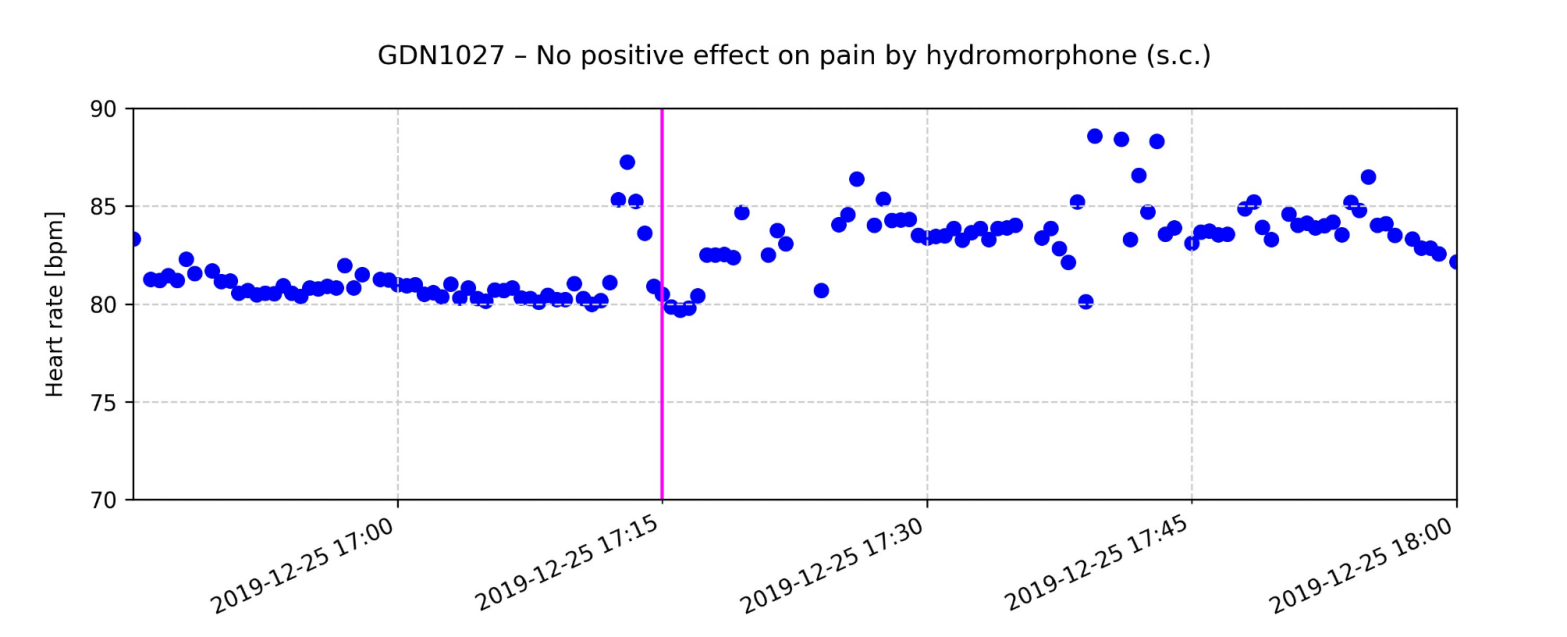
The pink line indicates the time of administration of 0.5 mg hydromorphone (s.c.) for pain. According to the CHR the patient reported to have no effect on symptom burden

The CHR-correlated HRs repeatedly revealed the potential of radar-based monitoring as shown by the following examples. The following graphs exemplarily show the effect of drug administration on the HR for selected patients (Figs. 6, 7 and 8).

Fig. 6 displays the course of HR over time of a patient who reported pain and nausea as documented in the CHR. At 06:40 h the average HR of the patient starts to rise from 75 bpm by approximately 10 bpm, reaching a peak well above 85 bpm. After administration of hydromorphone 1 mg, s.c. and Dimenhydrinat 62 mg, i.v. at 07:10 h the HR started to decrease reaching a plateau of approximately 75 bpm, which is in line with the onset and maximum of the expected drug effect. CHR reflected a relief of pain and nausea by a reduction of self-reported symptom severity on the MIDOS_2 scale by two points.

Fig. 7 shows the HR of a patient who reported dyspnoea. Hydromorphone 0.5 mg, s.c. was administered at 23:40 h. At that time, the patient’s radar-estimated HR was between 100 and 105 bpm. After drug administration the patient reported a positive effect on symptom burden as reflected in the CHR. The patient’s HR decreased by 5 to 10 bpm to approx. 95 bpm.

Fig. 8 shows the HR of a patient who reported pain. Hydromorphone 0.5 mg, s.c. was administered at 17:15 h. At that time, the patient’s radar-recorded HR was approximately 80 bpm. According to the CHR, the patient reported no relief of pain after administration. No decrease in HR after administration of the on-demand medication was observed. Instead, the HR increased.

## Discussion

In our study, we present the first approach of a burden-free radar-based approach to cardiac monitoring in PC patients and in a clinical PC environment. Our results show that radar-based HR monitoring is a feasible and useful approach in this setting. The radar-based system showed high accuracy compared to ECG, reflecting the possibilities and effectiveness of modern continuous cardiac monitoring. The results show minimal average differences and high agreement between the two methods.

For both overnight (study arm I) and one-hour (study arm II) measurements, we demonstrated a strong correlation between the HR determined by the radar system and the gold standard, i.e. ECG. Through statistical analysis, we established the equivalence of both methods. The modified Bland-Altman analysis and the equivalence test (TOST) further confirmed that the radar-based system’s measurements are within acceptable limits compared to ECG.

This underscores that non-invasive radar-based monitoring can reliably track HR in PC patients without burdening patients and providing physicians with important physiological data.

### Limitations and challenges

HR can be assessed by radar-based recording of microscopic movements caused by heart sounds in the micrometre range. Movement is a hallmark of life, and various movements such as breathing, turning or speaking overlay the vibrations caused by the heart sounds with much greater amplitudes. Furthermore, the location of radar measurement on the body can influence the HR estimation [33]. This can lead to wrong HR estimation by radar systems in a real-life PC setting. In our study, the validation recording was compromised by the same phenomena as both the Holter ECG and TFM signals were also distorted by movement. In study arm II, we excluded sections of poor ECG signal as we were lacking ground truth for validation. As a result, we may have overestimated the performance of radar-based HR recording in contrast to unlimited continuous measurement. In study arm I, no such sections were excluded to depict a real-life scenario. Nevertheless, the results obtained in study arm I and II are comparable.

Furthermore, various pathophysiological changes may occur in PC patients reducing the reliability of radar-based HR recording by our solution proposed in this work. For once, arrhythmia poses a challenge as the algorithms have not been trained on arrhythmia data. During the analysis of the radar data, we observed that estimating the HR from radar data during extra systoles and other arrhythmias is challenging for the HSMM. One reason may be that the HSMM initially estimates an average HR as a basis for subsequent segmentation. However, during arrhythmias, the HR may change abruptly and greatly from this segment’s average HR, which might not be adequately captured with the HSMM.

In addition, the HSMM depends on the correct segmentation of the first and second heart sound for HR estimation, as discussed above. However, during extra systoles and other types of arrhythmias the heart sounds are likely to be altered, which might compromise the heartbeat detection by the HSMM. Therefore, we decided to also label and exclude patient data with arrhythmic heart beats. To mitigate this issue, we are currently developing and evaluating an improved machine learning pipeline which might be able to solve these issues in the near future.

Another limitation may be pathophysiological changes of the heart’s muscular function towards the end of life: As our HR recording relies solely on the heart sounds, changes in inotropy and dromotropy may affect the heart sounds regarding frequencies and amplitudes. Whereas this poses a challenge in assessing the HR in PC patients, it may also hold a chance for radar monitoring to investigate the heart function beyond just chronotropy compared to ECG-based HR estimation. Further research is needed to determine possible effects of the heart muscle’s strength and dysfunction on radar-based heart sound recording.

Selecting the best-performing radar module out of 4 (study arm I and III) and out of 3 (study arm II), respectively was a challenge. The radar module with the highest SNR ratio, as reflected in the SQI, was chosen in study arms I and III. However, during the analysis of the one-hour measurements, we frequently observed that the radar system with the highest SQI does not necessarily deliver a HR that is closest to the ECG-derived HR. Therefore, the SQI as a singular measure for signal quality is insufficient. In study arm II, choosing the radar module with a HR that is closest to the ECG’s based on the beat-to-beat synchronization outperformed the module with the highest SQI. Therefore, further research is needed to determine features which can help identifying the most reliable radar-based HR signal solely from the radar-data.

### Summary and Outlook

Continuous HR monitoring in PC can provide invaluable information about a patient’s condition, particularly with regard to symptom management. Radar-based detection of HR and its changes can serve as an early and objective indicator of discomfort, pain or other distressing symptoms and allow timely and appropriate action to be taken. The documented effects of medication on HR, as shown in Figs. 6, 7 and 8, illustrate how physiological data can inform and optimize symptom control strategies.

The results of this study demonstrate significant potential for future applications in a clinical setting. The system is highly scalable in terms of its potential applications. From the perspective of patient care, continuous HR recording can facilitate the interpretation of clinical observations in patients who are unresponsive. As illustrated in the HR trajectories of patients with and without symptom improvement, there is a correlation between HR and symptom severity. Further research into radar systems may enable more precise and robust measurement, allowing the recording of heart rate variability (HRV) as a reliable indicator of well-being or stress. Changes in HR and associated features, such as HRV, as well as patterns of the radar-detected heart sounds, may be correlated with the deterioration of heart function towards the end of life. Such findings may therefore be used as prognostic biomarkers. There are numerous potential applications beyond PC. One potential application is a radar-based alarm system for cardiac complications and indications for cardio-pulmonary resuscitation in normal hospital wards or nursing homes. This could be a valuable addition to current care practices, as the radar systems require no additional personnel to start and carry out their measurement, and do not disturb or disrupt the patients’ lives on the ward. Further research must address the refinement of the technology and its feasibility, as well as the legal aspects of radar-based vital parameter monitoring.

Although radar technology can penetrate bedding and clothing, patient movement, pathophysiological changes, and external disturbances can introduce artefacts. Future developments in radar technology and signal processing algorithms are needed to enhance the system’s ability to differentiate between true cardiac signals and noise.

The study’s sample size, while adequate for initial validation, is small and the a priori calculated sample size for study arm II could not be reached due to recruitment limitations. Future studies should expand the sample size and diversity to validate the findings across various PC settings and patient demographics. This also enables the causes of outliers to be investigated further, e.g. an unfavourable position of the radar modules in relation to the patient’s position in bed or significant random body movements.

Additionally, advancing the technology to overcome limitations, to minimize artefacts and enhance signal accuracy will further solidify its utility in clinical practice. Investigating the integration of radar-based monitoring with other physiological parameters could provide a more comprehensive assessment of patient well-being.

Traditional monitoring methods run contrary to the fundamental principles of PC that emphasize comfort, care, and dignity. The radar-based approach offers a non-intrusive alternative, allowing for the unobstructed monitoring of HR without compromising patient comfort or privacy. Its non-intrusive nature is particularly beneficial in PC settings where the maintenance of a serene and non-clinical environment is paramount. Nevertheless, privacy concerns and data security related to radar-based continuous monitoring will have to be addressed. Therefore, further research into the social and ethical implications is necessary.

## Conclusions

Radar-based HR monitoring presents a promising, non-intrusive method for continuous physiological assessment in PC. Its implementation can enhance symptom management and improve patient care by providing accurate and reliable data without disrupting the patient’s environment. Continued research and technological advancements will be essential to fully realize the potential of this innovative approach in enhancing PC practices.

## Data Availability

The data that support the findings of this study are not openly available due to reasons of privacy and sensitivity and are available from the corresponding author upon reasonable request. Data are located in controlled access data storage at the University Hospital Erlangen.

## Abbreviations

bpm: Beats per minute
CHR: Clinical Health Record
ECG: Electrocardiography
HR: Heart Rate
HSMM: Hidden Semi-Markov Model
IBI: Inter-Beat Interval (Temporal distance between two R-peaks in the electrocardiogram)
ICG: Impedance Cardiography
i.v.: intravenous
s.c.: subcutaneous
SNR: Signal-to-Noise Ratio
SQI: Signal Quality Index
SVES: Supraventricular Extra Systole(s)
TFM: Task Force^®^ Monitor
TOST: Two One-Sided Test
VES: Ventricular Extra Systole(s)
