# Correction: contactless radar-based heart rate Estimation in palliative care– a feasibility study and possible use in symptom management

**DOI:** 10.1186/s12904-025-01741-2

**Published:** 2025-04-22

**Authors:** Stefan G. Grießhammer, Anke Malessa, Hui Lu, Julia Yip, Julie Leuschner, Florian Christgau, Nils C. Albrecht, Marie Oesten, Thanh Truc Tran, Robert Richer, Maria Heckel, Bjoern M. Eskofier, Alexander Koelpin, Tobias Steigleder, Christoph Ostgathe

**Affiliations:** 1 Department of Palliative Medicine, Universitätsklinikum Erlangen, Friedrich-Alexander-Universität Erlangen-Nürnberg (FAU), Erlangen, Germany; 2 Institute of High-Frequency Technology, Hamburg University of Technology (TUHH), Hamburg, Germany; 3 Chair of Electronics and Sensor Systems, Brandenburg University of Technology Cottbus-Senftenberg, Cottbus, Germany; 4 Machine Learning and Data Analytics Lab, Friedrich-Alexander- Universität Erlangen-Nürnberg (FAU), Erlangen, Germany

**Correction: BMC Palliat Care 23**,** 273 (2024).**

10.1186/s12904-024-01592-3

Following publication of the original article [1], the author reported that in the Abstract, Result section, the sentence:

“The HR difference was within +/-5 bpm for 53,583 out of 57,048 beats (93.93%) and within +/-8.2 bpm (± 1.96 times the SD of the mean) in 55,439 beats **(97.25%)**, respectively.”

Should be.

“The HR difference was within +/-5 bpm for 53,583 out of 57,048 beats (93.93%) and within +/-8.2 bpm (± 1.96 times the SD of the mean) in 55,439 beats **(97.18%)**, respectively.”

The calculation 55,439/57,048*100% equals to 97.18% and NOT as written 97.25%.

The original article has been updated.
